# Light management with quantum nanostructured dots-in-host semiconductors

**DOI:** 10.1038/s41377-021-00671-x

**Published:** 2021-11-17

**Authors:** M. Alexandre, H. Águas, E. Fortunato, R. Martins, M. J. Mendes

**Affiliations:** i3N/CENIMAT, Department of Materials Science, Faculty of Science and Technology, Universidade NOVA de Lisboa and CEMOP/UNINOVA, Campus de Caparica, 2829-516 Caparica, Portugal

**Keywords:** Nanophotonics and plasmonics, Quantum optics

## Abstract

Insightful knowledge on quantum nanostructured materials is paramount to engineer and exploit their vast gamut of applications. Here, a formalism based on the single-band effective mass equation was developed to determine the light absorption of colloidal quantum dots (CQDs) embedded in a wider bandgap semiconductor host, employing only three parameters (dots/host potential barrier, effective mass, and QD size). It was ascertained how to tune such parameters to design the energy level structure and consequent optical response. Our findings show that the CQD size has the biggest effect on the number and energy of the confined levels, while the potential barrier causes a linear shift of their values. While smaller QDs allow wider energetic separation between levels (as desired for most quantum-based technologies), the larger dots with higher number of levels are those that exhibit the strongest absorption. Nevertheless, it was unprecedently shown that such quantum-enabled absorption coefficients can reach the levels (10^4^–10^5 ^cm^−1^) of bulk semiconductors.

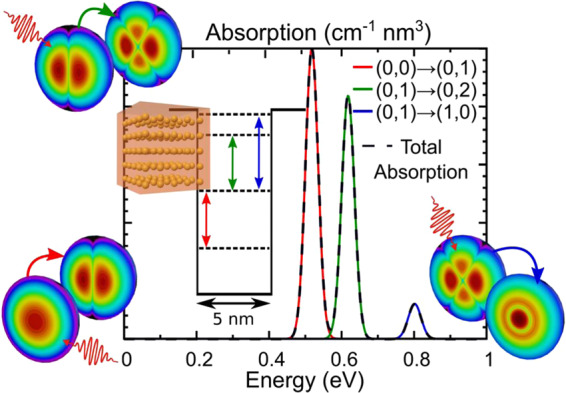

## Abstract (web summary)

Development of a simple yet insightful model to understand the optical behavior of dots-in-host semiconductors. Unprecedentedly shown that quantum-enabled absorption coefficients can reach levels close to that of bulk semiconductors.

## Introduction

The notable set of properties conferred to quantum dots (QDs) due to their nanoscale size has brought significant attention to their research and application^1–4^. They have been used effectively and flexibly in many different technologies, from device-oriented cases, such as infrared LEDs^3,5–7^, photovoltaics^8–12^, and luminescent down-shifting^13,14^, to applications in the biomedical and pharmaceutical industries, such as DNA hybridization and visualization of tissue and cellular structures in real time^15^, and even to quantum computing^16^. This interest is brought forth by the exceptional optoelectronic properties of such semiconductor nanoparticles, mainly resulting from their easily-tunable narrow-band absorption and/or emission^4,5,8,15^. These properties can also be entwined with other well-established technologies, as light-trapping in solar cells^17–20^, to go beyond their conventional (classical) limits^21^. Nonetheless, insightful fundamental studies are necessary to better understand the QDs response when integrated in different media, and allow researchers to fully utilize the vast gamut of quantum-enabled properties provided.

**Fig. 1:**
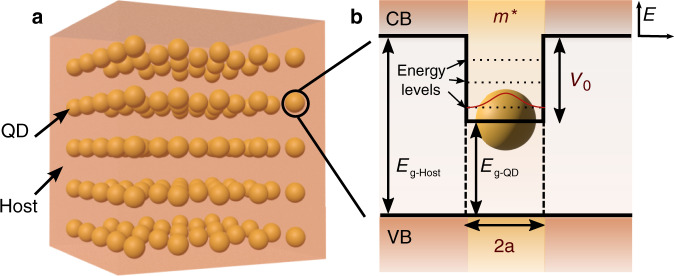
3D sketch and energy level diagram of nanostructured (dots-in-host) semiconductor. **a** Representation of an array of colloidal QDs, with radius *a* and effective mass m*, embedded in a host material (e.g., PbS QDs in a Perovskite host, as analyzed in this work). **b** Energy band diagram for the system in (**a**), where CB and VB represent, respectively, the conduction and valence band of the host, and *V*_0_ is the potential barrier given by the difference between the bandgaps of the host (*E*_G-Host_) and QD (*E*_G-QD_) materials. On the top right corner, we show the coordinate system used, in which the zero energy is set at the CB minimum

In this study, we developed a one-electron single-band effective mass formalism to evaluate the confined energy levels and resulting optical absorption of nanostructured semiconductors, i.e., host materials impregnated with QD arrays (Fig. 1)^22^. A spherical shape was considered for the dots, first because it has the highest symmetry and, second, it is the shape of colloidal QDs (CQDs) that have seen a surge in R&D interest^3,4,8,9,12^. Furthermore, to provide explicit calculations of the key parameters, a well-defined case was chosen by considering the QDs@host materials to be PbS@Perovskite, as this combination has been recently shown to form high-quality nanostructured semiconductors due to the spontaneous epitaxial-alignment established between the PbS CQDs and the Perovskite semiconductor^2,6,11,23^.

Here, the authors first study the behavior of the main parameters of the QDs (potential barrier between host and QDs, QDs radius, and effective mass) and their importance on the final optoelectronic properties. Extensive sweeps were subsequently performed to assess the inter-parameter influence on the ground-state energy level. Last, two QD radii (1.6 and 2.5 nm) were selected as examples due to their interest for realizing the intermediate-band solar cell (IBSC) concept^24–26^, and the energy levels and wavefunctions were determined to calculate the absorption coefficient for the nanostructured materials.

Our findings reveal that the QD radius has the single most important effect on the number and value of the energy levels, while the potential barrier is mostly responsible for shifting these values. It was also determined that the absorption coefficient depends on the separation between the levels, so that bigger QDs tend to have higher absorption peaks. Moreover, the QDs with 1.6 and 2.5 nm radius were shown to have only 1 and 3 allowed transitions, respectively, when confined in a 1.90 eV potential barrier. This result is highly desirable for applications that require as few energy levels as possible, such as IBSCs, where many unwanted transitions can lead to significant performance (voltage) losses for the devices.

## Results

We now discuss the results obtained from the developed single-band effective mass equation (SBEQ) formalism. To obtain a quantitative analysis we chose as variables for the problem a PbS (*E*_*G*_ = 0.40 eV)^27,28^ QD in a Perovskite host (average refractive index, *n*, of 2.5)^29^ with *E*_*G*_ of either 2.30 or 1.55 eV^30,31^. While the latter bandgap (1.55 eV) corresponds to that of state-of-the-art Perovskite solar cells, the former is the ideal for the intermediate-band solar cell (IBSC). The theoretical optimum IBSC absorber is a 2.30 eV bandgap host embedded with lower-bandgap QDs having a single level (the ground-state, *E*_0_), within the dots/host potential barrier (*V*_0_), placed at −0.90 eV from the minimum of the host CB^24^. Another determinant factor in this choice of semiconductors was the aforementioned excellent interfacial properties between these dots-in-host materials due to a high-quality lattice matching^2,6,32^. For this PbS CQDs-in-Perovskite study case the effective mass (m*) and potential barrier become respectively established as 0.08*m*_e_ (PbS’s effective mass^33–35^) and 1.90/1.15 eV (since *V*_0_ = *E*_G-Perovskite_ *−* *E*_G-PbS_). Unless otherwise stated these are the values used to determine the calculated properties, which are summarized in Table 1.

**Table 1:** Summary of the main properties of the PbS CQDs and Perovskite host, namely the effective mass, m*, bandgap, *E*_G_, potential barrier, *V*_0_, and refractive index, *n*, used for the simulations

	m* (*m*_e_)	*E*_*G*_ (eV)	*n*	*V*_0_ (eV)
PbS	0.08	0.4	–	1.9/1.15
Perovskite	–	2.3/1.55	2.5	

*V*_0_ was determined from the PbS’s and Perovskite’s bandgap (*V*_0_ = *E*_G-Perovskite_ – *E*_G-PbS_)

### Designing the energy levels diagram

**Fig. 2:**
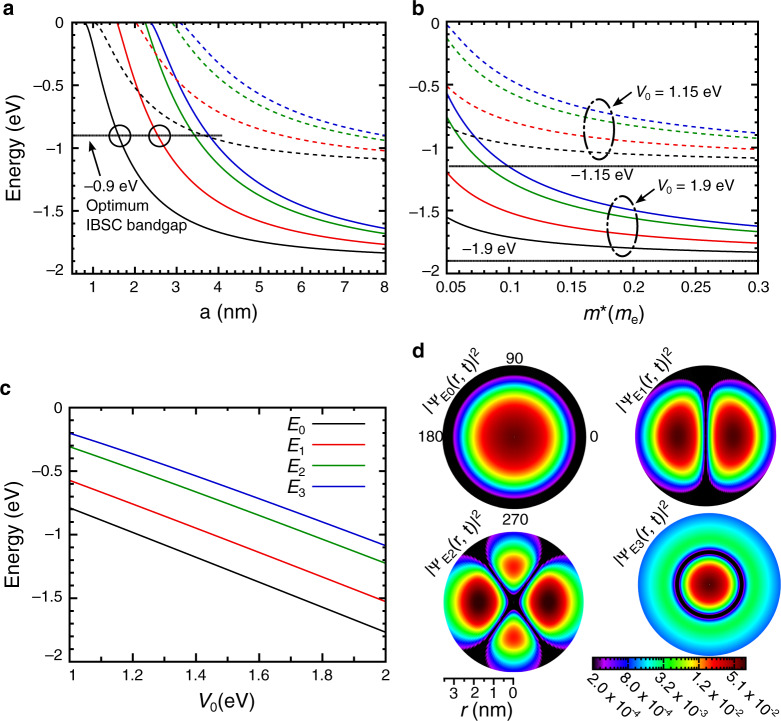
QD size impact on the first 4 energy levels (labeled from *E*_0_ to *E*_3_), the results were obtained with m* of 0.08*m*_e_. **a** QD size influence on the energy levels for potential barrier of 1.15 and 1.90 eV, dashed and continuous lines, respectively. The horizontal gray line indicates the ideal bandgap for an intermediate-band solar cell and the circles refer to the QD sizes used for the following studies. **b** QD effective mass influence on the energy levels for potential barrier of 1.15 and 1.90 eV, dashed and continuous lines, respectively. The horizontal gray lines represent the energy levels for −1.15 and −1.90 eV. **c** Effect of the potential barrier between the host and QD material on the energy levels. **d** Representation of the normalized wavefunctions for the first four energy levels of a 2.5 nm QD with 0.08*m*_e_ effective mass and 1.90 eV potential barrier, where the *m* value of the spherical harmonics was taken to be 0

We start by analyzing the SBEQ solutions and how they are impacted by the key factors involved. A unique effective mass was used for both materials to guarantee that the SBEQ remains Hermitian^22^. This is a reasonable simplification since the QD’s radius effect on the energy levels far outweighs that of the effective mass, as evidenced in the plots of Fig. 2. For example, from Fig. 2b one can see that, for *V*_0_ of 1.90 and 1.15 eV, changing m* from 0.05*m*_e_ to 0.30*m*_e_ has a ~17% impact on the ground state. Moreover, in the spherical well problem, the host’s effective mass does not create any new levels and thus its impact on the absorption profiles would not be significant^36^.

Figure 2a shows the first four energy levels (*E*_0-3_) in the QD, together with an horizontal line marking the ideal position (−0.90 eV^25^) of the confined levels forming the intermediate mini-band in the IBSC concept^24^. The intersection between this line and the *E*_0_ and *E*_1_ curves occurs for the radii of 1.6 and 2.5 nm, respectively, which will be used later for the calculation of the absorption coefficient. The 2.5 nm radius is also used as an example for the normalized probability density shown for the first 4 levels in Fig. 2d. The most notable aspects are (1) the delocalization of the electron’s wavefunctions in the QD, and (2) the effect of the centrifugal potential, that pushes the electron away from the QD center. These effects are common results from an analysis of the Schrödinger equation in a spherical system^36–38^, thus underlining the validity of the results.

As referred above, the QD radius has a strong impact on the energy levels. Figure 2a shows the steep exponential influence of the radius on the levels, most notably for smaller radii where even a marginal change in this value can severely affect the final energy level distribution. Moreover, the radius has a similar impact on all energy levels, in contrast with the effective mass, where the lower levels are less impacted by changing m*. Regarding the potential barrier (Fig. 2c), its variation causes a linear shifting of the levels, regardless of the other specific parameters. This is further emphasized in Fig. 2a, b, that show the results for two values of the potential barrier (*V*_0_ = 1.15 or 1.90 eV), demonstrating that the levels are simply shifted up/down by the same energy difference of the *V*_0_ values considered. This result is understandable, as the potential barrier defines the lower energy limit for the confined levels.

The QD shape is another important factor that impacts the energy levels of the system. However, from group theory considerations^39^ the shape most notably impacts the degeneracy of the energy levels, so that in lower symmetry shapes the degenerate energy levels break apart into several distinct levels^22,26^. Thus, a highly symmetric system is beneficial in situations where it is necessary to minimize the number of levels inside the QD.

**Fig. 3:**
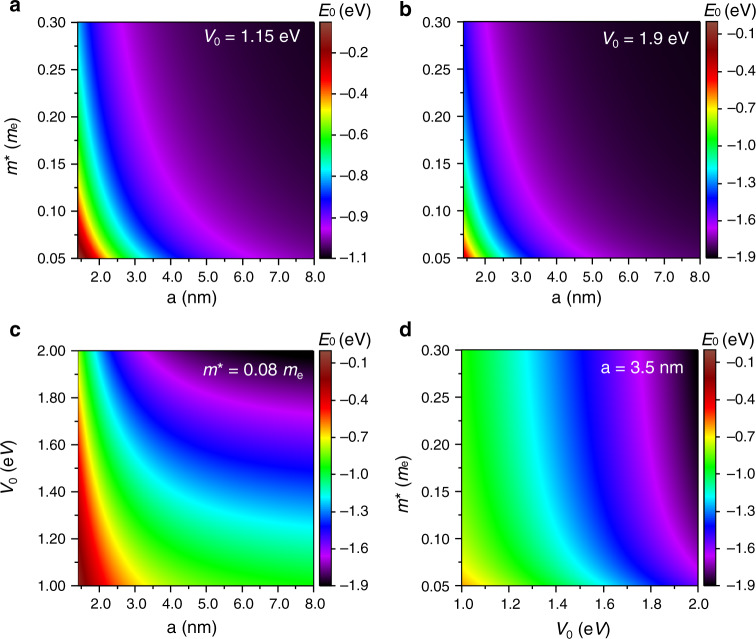
Inter-parameter relations for several combinations of QD properties, calculated for the ground-state energy level, *E*_0_. **a** Relation between the QD effective mass (m*) and radius (*a*) for a potential barrier (*V*_0_) of 1.15 eV. **b** Relation between the QD effective mass and the radius for *V*_0_ of 1.90 eV. **c** Relation between the potential barrier and the QD radius. **d** Relation between the QD effective mass and potential barrier, where a 3.5 nm QD radius is considered to guarantee at least one confined energy level in the full range of the profile

After assessing how the basic parameters can influence the QDs energy levels it is crucial to understand how they influence each other. Hence, extensive sweeps were performed to determine the inter-parameter relations for the ground-state level (*E*_0_) of the system (Fig. 3). These profiles can be seen as a super set of those shown in Fig. 2 for *E*_0_, thus complementing and providing a broader analysis of the parameter dependencies. In order to always guarantee the existence of at least one energy level, the minimum radius considered was 1.4 nm (value determined from the equation $\hbar \pi /\sqrt {8m_1V_0}$, that can be deduced from Equation S4 of Supplementary Material), while the fixed parameter in Fig. 3d was kept at 3.5 nm.

Figure 3a, b shows the relations between the effective mass and radius for the two potential barrier values (1.90 and 1.15 eV). These profiles clearly demonstrate the contrasting impact of the QD radius and effective mass on *E*_0_. This is mostly noticeable for smaller values of both QD radius and effective mass, where *E*_0_ has a steeper change when the QD radius is varied when compared with the effective mass. Moreover, for bigger QDs the impact of the effective mass on *E*_0_ also decreases. This constrained influence of the effective mass on the energy levels can be seen by taking the example of 3.5 nm radius, where the effective mass is shown to already have a diminished impact on *E*_0_, and is evident in Figure 3d that has mostly a horizontal (*V*_0_) dependence on *E*_0_. It can also be seen, when both Fig. 3a, b are combined, that for radii above 5 nm, *E*_0_ becomes close to the limit imposed by the potential barrier and is thence mostly unaffected by any change in either of the three parameters. This is further emphasized in Fig. 3c, where for radii above 5 nm *E*_0_ barely changes with the radius and its value is always close to its counterpart in *V*_0_.

These results underline that, for practical implementations, it is paramount to guarantee a monodispersion of the QD sizes, specially for smaller QDs, if a uniform set of properties is required.

### Quantum-enabled light absorption

**Fig. 4:**
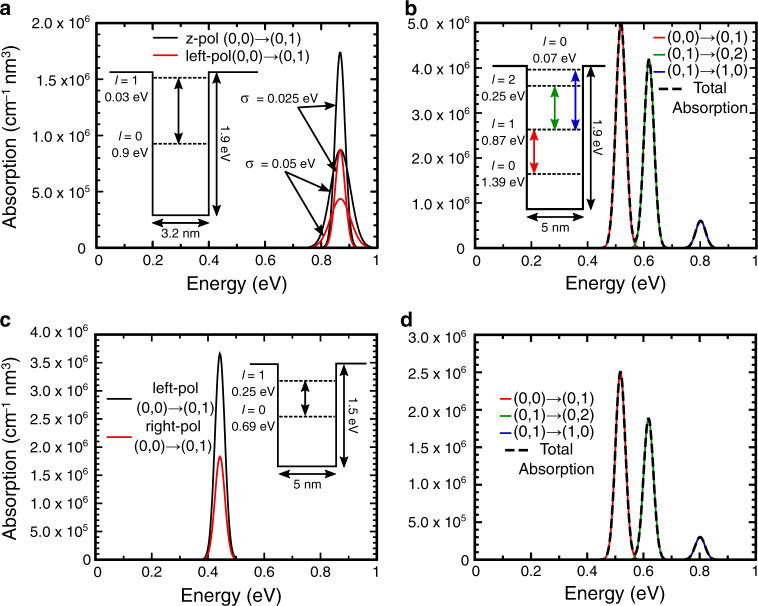
Absorption coefficient per density (*α*/*ρ*_qd_) spectra for different QD properties. The inset schematics are the energy band diagrams for each case, showing the allowed transitions. **a** Spectrum for a 1.6 nm QD with *V*_0_ of 1.90 eV, for z and left polarizations (black and red profile, respectively), and considering two gaussian dispersions, *σ*, of 0.025 and 0.05 eV (full and dotted lines, respectively). **b**, **d** Profiles for a 2.5 nm QD with *V*_0_ of 1.90 eV, for z and left polarizations, respectively. The red, green, and blue profiles represent each allowed transition. **c** Profile for a 2.5 nm QD with *V*_0_ of 1.15 eV for z and left polarizations (black and red profile, respectively)

The absorption spectra are now determined with the method described in the Methods Section 2, using the values in Table 1. The results are shown in Fig. 4 for 2 QD radii and 2 potential barriers: 1.6 nm/1.90 eV (Fig. 4a), 2.5 nm/1.90 eV (Fig. 4b, d), and 2.5 nm/1.15 eV (Fig. 4c). The absorption for the 1.6 nm/1.15 eV QD was not possible to calculate as there was only a single energy level present within the barrier, while at least two states are needed to allow a photon-induced electronic transition. For comparison of the results, in Supplementary Material Section S4 we provide additional absorption profiles for the QD radii (2.5 and 3.9 nm) that also result in an energy level at −0.9 eV but for a potential barrier of 1.15 eV, and with a higher effective mass of 0.17*m*_e_ (as the literature reports for this value fluctuate between 0.08*m*_e_ and 0.17*m*_e_)^33–35,40^.

In any dots-in-host material (see Fig. 1), the higher the volumetric density of QDs in the array the higher the absorption enabled by their confined states. This is accounted for in Eq. (6) with the proportionality term corresponding to the QD density in the host matrix, *ρ*_qd_. To allow the applicability of the results of Fig. 4 to any particular QD density, it is preferable to present the absorption coefficient per density (*α*/*ρ*_qd_ [cm^−1^ nm^3^]) as it can be multiplied afterward with the *ρ*_qd_ values to determine the specific absorption coefficient (*α* [cm^−1^]). For instance, CQDs can be densely packed in dots-in-host films with volumetric densities up to 10^19^–10^20 ^dots/cm^3 ^^24,41^. As such, taking the peak absorption coefficient densities attained in Fig. 4, it is possible to achieve absorption coefficients of 10^4^–10^5 ^cm^−1^ for the transitions, closely following recent experimental results of intra-CQD transitions by Ramiro et al. measured with QD densities of ~10^19^ cm^−3 ^^41^. Remarkably, these are values comparable to the bulk absorption coefficients of macroscopic semiconductors, which is a clear demonstration of the potentialities of quantum-structured materials for any optoelectronic application.

Another important aspect in practical applications is the QD size dispersion, which affects the absorption peaks, such that larger dispersions lead to wider and smaller peaks. In our model, this effect can be accounted for by varying the width of the gaussian (*σ*) used to approximate the delta function in Eq. (6). In the present study the authors used a value of *σ* = 0.025 eV—similar to other previous works^22,26^—however, Fig. 4a compares the calculation of the absorption coefficient of a 1.6 nm radius QD for gaussian widths of 0.025 and 0.05 eV (representing a broader QD size dispersion). As expected from an experimental standpoint, the wider size dispersion not only leads to a broader peak, but also to a reduction in the absorption coefficient maximum.

The most notable and expected contrast for the 1.6 and 2.5 nm radii is the number of energy levels, as the larger the QD the higher the number of levels. In a more practical context of quantum-based technologies, a higher number of levels can lead to different unwanted effects, such as higher number of allowed transitions. For instance, in IBSCs, thermal and tunnel-assisted carrier escape from the intermediate band (QD) to the conduction band (host) have been shown^42–44^, which entail non-radiative recombination that effectively hampers the achievement of photovoltage preservation (i.e., maximum *V*_oc_ chiefly limited by the wider host bandgap and not by the presence of the intermediate band), thus limiting the efficiency of the cells. In this respect, spherical QDs, as considered in this work, are advantageous—being the highest symmetry geometry it will have the highest degree of degeneracy, and thence a lower number of levels. For instance, a 2.65 nm cubical QD—size with the same volume as a 1.6 nm radius spherical QD—will have 7 non-degenerate energy levels in contrast with the 2 levels of the spherical QD. Furthermore, other works have also shown that 8.5 nm square QDs (equivalent in volume to a 3.3 nm spherical QD) have >15 energy levels, effectively doubling the energy levels from the 2.65 nm case^21^.

In Eq. (6), the angular components are responsible for defining the allowed transitions (see Supplementary Material Section S1). As shown in the inset schematics of Fig. 4, not all transitions between levels are allowed, which reduces the number of possibly unwanted transitions. These transition rules can be established by developing the matrix elements, ${\left| {\left\langle {{{\Phi}_j}} \right|{\epsilon^{(\lambda )}} \cdot r\left| {{{\Phi}_i}} \right\rangle } \right|^2}$, that also include polarization dependency. Here, the model benefits from using spherical coordinates by expanding ${\it{\epsilon }}^{\left( \lambda \right)} \cdot r$ in terms of spherical harmonics (Equation S8 of Supplementary Material) to ultimately separate Eq. (6) into three different equations (Equation S9 of Supplementary Material) that depend on three different polarizations, z, left circularly polarized (LCP) and right circularly polarized (RCP). Thence, it is possible to separately determine the absorption for specific polarizations. Note that the z polarization represents an electric field changing in the light-propagation direction while both the left and right polarizations simply represent a combination of the x and y-polarizations that would normally appear in a cartesian problem. For this case, each polarization term is thus responsible for defining a set of rules that establish the allowed transitions that can be summarized as ${\Delta}l = \pm 1,m_f - m_i = \pm 1$^37,45^. Therefore, when a transition between 2 levels occurs, the initial and final *m* values can also impact the transition. However, this is a degenerate level conscious rule, i.e., it only affects the transitions between degenerate levels, and does not really impact the number of transitions. As such, to simplify the problem, these transitions between degenerate states were considered to be equally likely, and thus the overall transitions probability was taken as the average of all these transitions^22^. Nevertheless, since different polarizations also contribute with different angular matrix elements, and consequently different absorption coefficient peaks, the authors also provide the results for the z and LCP polarizations (the RCP results are equal to the LCP’s). It should nevertheless be emphasized that these absorption coefficient peaks correspond to transitions between different degenerate states of 2 energy levels. Figure 4 clearly shows that the QDs have higher absorption for the z polarization, simply because the matrix elements, as determined in Supplementary Material Section S3, are higher for the z component than for the LCP and RCP.

The equations describing the angular and radial integrals, necessary to calculate the absorption profile, are provided in Supplementary Material Section S1 (Equations S7,S9,S10), and the calculated values for the case studied in this article are provided in Supplementary Material Sections S2 and S3 (Tables S1,S2,S3).

Last, the bigger QDs show higher intensity absorption peaks. The matrix elements from Eq. (6) are what ultimately defines such intensity. However, in contrast to the aforementioned z vs. left polarization disparity, here it is the radial integral the main responsible for such difference. From Supplementary Material Equation S7 this element is directly proportional to the overlap between the radial components of the wavefunction between the initial and final states. Therefore, generally the bigger the separation between 2 energy levels, the smaller the overlap between these 2 radial components. Figure 2d shows well this effect between the 1st and 3rd wavefunctions, as can further confirmed by a simple analysis of the spherical Bessel functions used to calculate the wavefunctions. In Fig. 4, the energy levels that are more separated result in smaller absorption peaks. Interestingly, this trend is also followed by the results of Ramiro et al.^41^, that show a reduction in absorption peak intensity as the peak center moves to higher energies and higher absorption coefficients for bigger QDs.

It should also be noted that there is a significant difference between the crystal structure symmetry and QD shape symmetry. For the former, the influence is mostly noticed in the symmetry of the wavefunctions^39,46^, i.e., the symmetry properties of the structure are reflected on the symmetry properties of the wavefunctions. This can be relevant for inter-band transitions^22^, where different symmetries from different bands can influence the transition probabilities, but not so significantly for the intra-band transitions which are the focus of this work. For the QD shape symmetry, the effect occurs mainly on the energy level degeneracy, as previously discussed, consequently its effect is most relevant on the number of allowed transitions.

## Methods

This work used the single-band effective mass equation (SBEQ, Eq. (1)) as a basis to study of the QDs properties^22,47^. In this approach, the full wavefunction as obtained from the time-independent Schrödinger equation (TISE), is shown to be approximately expressed as the product of a periodic function (representing the Block component) and an envolvent function, *Φ*(*r*), that fulfills Eq. (1) (detailed derivation provided in ref. ^47^). This envelope function can thence be used to determine the QD properties. Such method was chosen due to the insightful simplicity provided by this approach, contrasting with other more fundamental, however, also more abstract/complex formalisms^26,48^.

1 $$\frac{{ - \hbar ^2}}{{2m_\nu ^ \ast }}\nabla ^2{\Phi}\left( r \right) + \left( {E_{\nu ,0} + U} \right){\Phi}\left( r \right) = E{\Phi}\left( r \right)$$

In Eq. (1), *ħ* is the reduced Plank’s constant, *m*_*v*_ is the effective mass, (*E*_*ν,*0_*+U*) represents the potential barrier of the system, where *E*_*ν,*0_ is the local component and *U* the periodic component, *E* represents the allowed energy levels.

Subsequently, due to the strong interest in colloidal QDs (CQDs), a spherical potential was taken—used to represent *E*_*ν,*0_*+U* in Eq. (1)—as defined in Eq. (2), where *V*_0_ is the potential difference between the conduction band energies of the QD and host materials, as shown in Fig. 1, and *a* is the QD radius.

2 $$V\left( r \right) = \left\{ {\begin{array}{*{20}{c}} { - V_0} & {,r\le a} \\ 0 & {,r \,>\, a} \end{array}} \right.$$

The problem is thus reduced to a standard differential equation problem (the finite-spherical well) that has been widely studied^36–38,47^, and whose solution is detailed in Supplementary Material Section S1. Ultimately, the eigenvalue equation (Eq. (3) for *l* ≠ 0 and Equation S4 of Supplementary Material for *l* = 0) is reached by matching the logarithmic derivatives of the wavefunction inside and outside the QD. Equation (3) can then be solved numerically to obtain the set of energy levels of the embedded QD.

3 $$\sqrt {\frac{{m^{\ast} \left( {E + V_{0}} \right)}}{{m^{\ast} E}}} \frac{{j_{l - 1}\left( {\sqrt {\frac{{2m^{\ast} \left( {E + V_{0}} \right)}}{{{\hslash}^{2}}}} a} \right)}}{{j_{l}\left( {\sqrt {\frac{{2m^{\ast} \left( {E + V_{0}} \right)}}{{{\hslash}^{2}}}} a} \right)}} - i\frac{{h_{l - 1}^{\left( 1 \right)}\left( {i\sqrt {\frac{{\sqrt { - 2m^{\ast} E} }}{{{\hslash}^{2}}}} a} \right)}}{{h_{l}^{\left( 1 \right)}\left( {i\sqrt {\frac{{ - 2m^{\ast} E}}{{{\hslash}^{2}}}} a} \right)}} = 0$$

Where, *j*_*n*_ is the spherical Bessel function, *h*_*n*_ is the first order spherical Hankel function, *E* is the energy of a particular level and m* is the electron effective mass in the QD material. Here, we considered the effective mass to be spatially invariant to maintain the effective-mass equation Hermitian^22,47^. The normalized wavefunctions (Eq. (4)) can thence be determined by coupling the wavefunction normalization and continuity conditions (derivation shown in Supplementary Material Section S1).

4 $$\begin{array}{c} {\Phi}\left( {r,\theta ,\phi } \right) = \left\{\begin{array}{cc} A_nj_n\left( k_{{{{\mathrm{in}}}}}r \right)Y_l^m\left( \theta ,\phi \right) & ,r\le a \\ B_nh_n^{\left( 1 \right)} \left( ik_{{{{\mathrm{out}}}}}r \right) Y_l^m\left( \theta ,\phi \right) & ,r \, < a\end{array}\right.\\ A_l^2 = \frac{\left| h_l^{\left( 1 \right)}\left( k_{\mathrm{out}}a \right) \right|^2}{a\left| h_l^{\left( 1 \right)}\left( k_{\mathrm{out}}a \right) \right| + b\left| l_l\left( k_{\mathrm{in}}a \right) \right|^2}, B_l^2 = \frac{\left| j_l\left( k_{\mathrm{in}}a \right) \right|^2}{a\left| h_l^{\left( 1 \right)}\left( k_{\mathrm{out}}a \right) \right| + b\left| l_l\left( k_{\mathrm{in}}a \right) \right|^2} \end{array}$$

Where the abbreviations $ik_{{{{\mathrm{out}}}}} = \sqrt { - 2m^ \ast E/\hbar ^2}$, $k_{{{{\mathrm{in}}}}} = \sqrt {2m^ \ast \left( {E + V_0} \right)/\hbar ^2}$, $a = {\int}_0^a {j_l^2\left( {k_{{{{\mathrm{in}}}}}r} \right)r^2dr}$, $b = {\int}_a^\infty {h_l^{\left( 1 \right)^2}\left( {k_{{{{\mathrm{out}}}}}r} \right)r^2dr}$, and $Y_l^m\left( {\theta ,\phi } \right)$ are the spherical harmonics.

The optical absorption properties of the QDs can be determined by utilizing Fermi’s Golden Rule (Eq. (5)) using the dipole approximation, to calculate the transition rate, *Γ*_*i→j*_, between an initial, *Φ*_*i*_, and final state, *Φ*_*j*_^22,37,47^.

5 $${\Gamma}_{i \to j} = \frac{{\pi q^2\omega }}{{{\it{\epsilon }}_r{\it{\epsilon }}_0V}}\left|\langle {{\Phi}_j{{{\mathrm{|}}}}{\it{\epsilon }}^{\left( \lambda \right)} \cdot r{{{\mathrm{|}}}}{\Phi}_i} \rangle\right|^2\delta \left( {E_j - E_k \mp \hbar \omega } \right)$$

Where, *ω* is the angular velocity of the absorbed/emitted photon, *q* is the electron charge, *V* is the crystal volume, *ε*_0_ is the vacuum permittivity, *ε*_*r*_ is the medium’s dielectric constant, *ε*^*(λ*)^ is the polarization vector, and *E*_*j*_ and *E*_*i*_ are the energies of the final and initial state, respectively. This transition rate is then used to determine the absorption coefficient, *α* of the QD as follows (Eq. (6)).

6 $$\alpha _{i \to f} = \frac{{2\pi q^2E}}{{n_{{{{\mathrm{ref}}}}}ch{\it{\epsilon }}_0}}\left| \langle{{\Phi}_j{{{\mathrm{|}}}}{\it{\epsilon }}^{\left( \lambda \right)} \cdot r{{{\mathrm{|}}}}{\Phi}_i} \rangle\right|^2\rho _{{{{\mathrm{qd}}}}}\delta \left( {E_f - E_i - \hbar \omega } \right)f_i\left( {1 - f_f} \right)$$

Where *n*_ref_ is the refractive index of the host material, *E* is the energy of the absorbed photon, *c* is the light velocity in vacuum, *ρ*_qd_ is the QDs volumetric density in the array, and *f*_*i*_ and *f*_*f*_ are the Fermi factors for the initial and final state, respectively. These later values represent the occupancy of a particular state, so a Fermi factor of 1 for the final state represents a null absorption coefficient regarding that particular transition, as that state is fully occupied. For simplicity of the analysis, here the initial state is taken to be fully occupied and the final state is fully empty (*f*_*i*_ and *f*_*f*_ equal to 1 and 0, respectively). We also note that the matrix element for the transition $\left\langle {{\Phi}_j|{\it{\epsilon }}^{\left( \lambda \right)} \cdot r|{\Phi}_i} \right\rangle$ has to be considered carefully, as it will be separated into three different components (z polarization, left circular polarization, LCP, and right circular polarization, RCP) from the angular integration (a detailed development, and the final expression used for this calculation is shown in Supplementary Material S1). Last, Dirac’s Delta function (*δ*) is a sharply defined peak at energy *E*_*f*_*–E*_*i*_, however, small variations of the parameters can lead to a broadening of this peak, and thus this function can be approximated by a Gaussian profile. In this work, the peak broadening was maintained at 0.025 eV, similar to other previous works^22^.

## Conclusions

The formalism developed in this study benefits from its inherent simplicity, flexibility, and results accuracy, in contrast with other significantly more complex methods, making it thus an ideal choice for designing quantum-structured semiconductors.

Several properties of spherical QDs and their impact on the energy levels were investigated, namely the radius, effective mass, and potential barrier. It was ascertained that the size has the most relevant impact on the number and value of the confined levels. The potential barrier was determined as the main factor for determining the magnitude of the energy levels as it defines the limit value that these can have. Last, the effective mass mainly affects the higher energy levels.

The light interaction properties of the dots-in-host systems were then studied by calculating the absorption coefficient per density (*α*/*ρ*_qd_) for QD radii and potential barriers of interest, where it was determined that the bigger sizes had more allowed transitions and higher absorption. This latter effect was associated with a smaller separation between the energy levels that provide better coupling between both states, and thus increases the absorption coefficient. Last, it was determined that QD densities (~10^19 ^cm^−3^) attainable by CQDs can result in absorption coefficients comparable to those of standard photovoltaic materials, thus revealing the remarkable potentialities for future technologies based on quantum-structured semiconductors.

## Supplementary information

Supplementary Material

## References

[1] Kroupa DM (2017). Tuning colloidal quantum dot band edge positions through solution-phase surface chemistry modification. Nat. Commun..

[2] Ning ZJ (2015). Quantum-dot-in-perovskite solids. Nature.

[3] Shirasaki Y (2013). Emergence of colloidal quantum-dot light-emitting technologies. Nat. Photonics.

[4] Supran GJ (2015). High-performance shortwave-infrared light-emitting devices using core–shell (PbS–CdS) colloidal quantum dots. Adv. Mater..

[5] Gong XW (2016). Highly efficient quantum dot near-infrared light-emitting diodes. Nat. Photonics.

[6] Gao L (2020). Efficient near-infrared light-emitting diodes based on quantum dots in layered perovskite. Nat. Photonics.

[7] Sargent EH (2005). Infrared quantum dots. Adv. Mater..

[8] Kim T (2020). Efficiency limit of colloidal quantum dot solar cells: effect of optical interference on active layer absorption. ACS Energy Lett..

[9] Mendes MJ (2013). Self-organized colloidal quantum dots and metal nanoparticles for Plasmon-enhanced intermediate-band solar cells. Nanotechnology.

[10] Mendes MJ (2009). Plasmonic light enhancement in the near-field of metallic nanospheroids for application in intermediate band solar cells. Appl. Phys. Lett..

[11] Han JH (2018). Hybrid PbS quantum-dot-in-perovskite for high-efficiency perovskite solar cell. Small.

[12] Jeong KS (2012). Enhanced mobility-lifetime products in PbS colloidal quantum dot photovoltaics. ACS Nano.

[13] Alexandre M (2019). Optimum luminescent down-shifting properties for high efficiency and stable perovskite solar cells. ACS Appl. Energy Mater..

[14] Liu N (2018). ZnSe/ZnS core-shell quantum dots incorporated with Ag nanoparticles as luminescent down-shifting layers to enhance the efficiency of Si solar cells. J. Alloys Compound..

[15] Pawar, R. S., Upadhaya, P. G. & Patravale, V. B. In *Handbook of Nanomaterials for Industrial Applications* (ed. Hussain, C. M.) 621–637 (Elsevier, 2018).

[16] Loss D, DiVincenzo DP (1998). Quantum computation with quantum dots. Phys. Rev. A.

[17] Haque S (2020). Design of wave-optical structured substrates for ultra-thin perovskite solar cells. Appl. Mater. Today.

[18] Mendes MJ (2016). Design of optimized wave-optical spheroidal nanostructures for photonic-enhanced solar cells. Nano Energy.

[19] Centeno P (2020). Self‐cleaned photonic-enhanced solar cells with nanostructured parylene‐C. Adv. Mater. Interfaces.

[20] Li KZ (2020). Light trapping in solar cells: simple design rules to maximize absorption. Optica.

[21] Luque A (2010). Intraband absorption for normal illumination in quantum dot intermediate band solar cells. Sol. Energy Mater. Sol. Cells.

[22] Luque, A. & Mellor, A. V. *Photon Absorption Models in Nanostructured Semiconductor Solar Cells and Devices* (Springer, 2015).

[23] Ngo TT, Mora-Seró I (2019). Interaction between colloidal quantum dots and halide perovskites: looking for constructive synergies. J. Phys. Chem. Lett..

[24] Ramiro I, Martí A (2020). Intermediate band solar cells: present and future. Prog. Photovolt. Res. Appl..

[25] Okada Y (2015). Intermediate band solar cells: recent progress and future directions. Appl. Phys. Rev..

[26] Luque A (2011). New Hamiltonian for a better understanding of the quantum dot intermediate band solar cells. Sol. Energy Mater. Sol. Cells.

[27] Nanda KK (2002). Band-gap tuning of PbS nanoparticles by in-flight sintering of size classified aerosols. J. Appl. Phys..

[28] Scanlon WW (1959). Recent advances in the optical and electronic properties of PbS, PbSe, PbTe and their alloys. J. Phys. Chem. Solids.

[29] Phillips LJ (2015). Dispersion relation data for methylammonium lead triiodide perovskite deposited on a (100) silicon wafer using a two-step vapour-phase reaction process. Data Brief.

[30] Brenner TM (2016). Hybrid organic–inorganic perovskites: low-cost semiconductors with intriguing charge-transport properties. Nat. Rev. Mater..

[31] Chapa M (2019). All-thin-film perovskite/C–Si four-terminal tandems: interlayer and intermediate contacts optimization. ACS Appl. Energy Mater..

[32] Hou Y (2020). Efficient tandem solar cells with solution-processed perovskite on textured crystalline silicon. Science.

[33] Dixon JR, Riedl HR (1965). Optical dispersion of lead sulfide in the infrared. Phys. Rev..

[34] Rabii S (1968). Investigation of energy-band structures and electronic properties of PbS and PbSe. Phys. Rev..

[35] Nanda KK (2004). Effective mass approximation for two extreme semiconductors: band gap of PbS and CuBr nanoparticles. J. Appl. Phys..

[36] Messiah, A. *Quantum Mechanics* (Dover Publications, 2014).

[37] Gasiorowicz, S. *Quantum Physics* 3rd edn (Wiley, 2003).

[38] Griffiths, D. J. & Schroeter, D. F. *Introduction to Quantum Mechanics* 2nd edn (Cambridge University Press, 2017).

[39] Dresselhaus, M. S., Dresselhaus, G. & Jorio, A. *Group Theory: Application to the Physics of Condensed Matter* (Springer, 2008).

[40] Walton AK, Moss TS, Ellis B (1962). Determination of the electron effective mass in the lead salts by the infra-red faraday effect. Proc. Phys. Soc..

[41] Ramiro I (2020). Size-and temperature-dependent intraband optical properties of heavily n-doped PbS colloidal quantum dot solid-state films. ACS Nano.

[42] Ramiro, I. et al. InAs/AlGaAs quantum dot intermediate band solar cells with enlarged sub-bandgaps. In *38th IEEE Photovoltaic Specialists Conference* (IEEE, 2012).

[43] Fry PW (2001). Photocurrent spectroscopy of InAs/GaAs self-assembled quantum dots. Phys. Status Solidi.

[44] Antolín E (2010). Reducing carrier escape in the InAs/GaAs quantum dot intermediate band solar cell. J. Appl. Phys..

[45] Arfken, G. B., Weber, H. J. & Harris, F. E. *Mathematical Methods for Physicists A Comprehensive Guide* 7th edn (Elsevier, 2013).

[46] Willatzen, M. & Lew Yan Voon, L. C. *The k p Method* (Springer, 2009).

[47] Datta, S. *Quantum Phenomena* (Addison-Wesley, 1989).

[48] Peng HW (2008). First-principles study on rutile TiO_2_ quantum dots. J. Phys. Chem. C.

