# Supplementary material for: Light management with quantum nanostructured dots-in-host semiconductors
Source: Light Sci Appl. 2021 Nov 17;10:231. doi: 10.1038/s41377-021-00671-x (PMC8595380; doi:10.1038/s41377-021-00671-x)
Supplement: Supplementary file 1 — Supplementary Material [file 41377_2021_671_MOESM1_ESM.docx]

Supplementary Material

Light Management with Quantum Nanostructured Dots-in-Host Semiconductors

M. Alexandre, H. Águas, E. Fortunato, R. Martins, M. J. Mendes

i3N/CENIMAT, Department of Materials Science, Faculty of Science and Technology, Universidade NOVA de Lisboa and CEMOP/UNINOVA, Campus de Caparica, 2829-516 Caparica, Portugal

*Corresponding authors: [m.alexandre@campus.fct.unl.pt](mailto:m.alexandre@campus.fct.unl.pt), [mj.mendes@fct.unl.pt](mailto:m.mendes@fct.unl.pt)

1. Mathematical Foundations

In this section the authors provide a detailed description for the solutions of the single band effective mass equation (SBEQ) in spherical quantum dots (QDs). Considering the potential used for this system (as shown in Main Manuscripts’ Equation 2), a change of coordinates in the SBEQ is necessary, leading to the following radial equation (Equation S1).

| $\frac{-\hbar}{2m^{\text{*}}}\left( \frac{d^{2}}{{dr}^{2}}+\frac{2}{r}\frac{d}{dr}-\frac{l\left( l+1 \right)}{r^{2}} \right)R_{nl}\left( r \right)+V\left( r \right)R_{nl}\left( r \right)=E_{nl}R_{nl}\left( r \right)$ | (S1) |
| --- | --- |

Where *V(r)* is defined from Equation 2 of the Main Manuscript, *R_nl_* is the radial wavefunction, *l* represents the angular momentum number and *m^*^* the effective mass for the electron. The angular part of the equation is not shown as it is the well-known standard equation leading to the spherical harmonics.^1,2^ The solutions to the problem are thus as follows (since the potential is defined partwise, so are the solutions).

| $R\left( r \right)=\left\{ \begin{matrix} A_{\text{in}}j_{l}\left( k_{\text{in}}r \right) & ,r\leq a \\ A_{\text{out}}h_{l}^{1}\left( {ik}_{\text{out}}r \right) & ,r>a \end{matrix} \right.$ | (S2) |
| --- | --- |

Here, *A_in_* and *A_out_* are the normalization constants, *a* is the QD radius, *j_l_* and *h_l_*^1^ are the spherical bessel and hankel functions of the first kind, respectively, and *k_in_* and k_out_ are defined as $k_{\text{in}}={\sqrt{2m^{\text{*}}\left( E+V_{0} \right)}}/\hbar$ and ${jk}_{\text{out}}={\sqrt{2m^{\text{*}}E}}/\hbar$. From here it is then necessary to apply the boundary conditions (in this case the logarithmic match of the derivative – Equation S3) to reach an equation that can be solved to obtain the eigenvalues (energy levels) – Equation 3 of the Main Manuscript.

| $k_{\text{in}}\left( \frac{\frac{dj_{l}\left( \rho\right)}{d\rho}}{j_{l}\left( \rho\right)} \right)_{\rho=k_{\text{in}}a}=ik_{out}\left( \frac{\frac{dh_{l}\left( \rho\right)}{d\rho}}{h_{l}\left( \rho\right)} \right)_{\rho=ik_{out}a}$ | (S3) |
| --- | --- |

Here, several approaches can be taken to solve the problem. Either the derivatives can be calculated by “brute force” with an iterative method, for instance using the central differences method; or instead, a simplification to these expressions can be made. As such, considering the following simplifications, ${{dh}_{l}}/d\rho=h_{l-1}-\left( l+1 \right)/\rho h_{l}$and ${{dj}_{l}}/d\rho=j_{l-1}-\left( l+1 \right)/\rho j_{l}$, it is preferential to simplify Equation S3 as this method also allows for more accurate results. As referred in the main manuscript, this simplification is only valid for l > 0, and as such the equation for l = 0 should be calculated separately. The equation for the generic case (l>0) is already provided in the Main Manuscript (Equation 3), and the equation for l = 0 is as follows.

| $-cot\left( \sqrt{\frac{2m_{1}\left( E+V_{0} \right)}{\hbar^{2}}}a \right)=\sqrt{\frac{-m_{2}}{m_{1}}\frac{-E}{V_{0}+E}}$ | (S4) |
| --- | --- |

From the previous equations it is possible to calculate the energy levels. However, the wavefunctions are also required to compute the absorption spectra. As such, the normalization constants need to be determined. For that purpose, 2 sets of conditions were used. Firstly, the normalization condition was used (Equation S5).

| $\int A^{2}j_{l}^{2}\left( k_{\text{in}}r \right)r^{2}dr+\int B^{2}h_{l}^{\left( 1 \right)^{2}}\left( k_{out}r \right)r^{2}dr=1$ | (S5) |
| --- | --- |

Subsequently, since the above equation does not entirely define the problem, an additional condition, obtained from the aforementioned boundary conditions was used to remove the ambiguity in the solutions.

| $Aj_{l}\left( k_{\text{in}}a \right)-Bh_{l}^{\left( 1 \right)}\left( k_{out}a \right)=0$ | (S6) |
| --- | --- |

From the above equations (Equation S5 and S6) it is then possible to obtain a full expression for the wavefunctions as shown in Equation 4 of the Main Manuscript.

Lastly, having defined the wavefunctions and energy levels, all the ingredients are thus available to calculate the absorption coefficient. For that, the starting point is Fermi’s Golden rule (Equation 5 of the Main Manuscript). From there, the matrix elements can be evaluated using the above obtained results (Equation S7).

| $\left\langle\Phi_{j} \vert\epsilon^{\left( \lambda\right)}\cdot\vec{r} \vert\Phi_{i} \right\rangle=\int r^{3}drR_{n_{f}l_{f}}^{\text{* }}\left( r \right)R_{n_{i}l_{i}}\left( r \right)\times\int d\Omega Y_{n_{f}l_{f}}^{\text{*}}\left( \theta,\phi\right)\epsilon^{\left( \lambda\right)}\cdot\hat{r}Y_{n_{i}l_{i}}\left( \theta,\phi\right)$ | (S7) |
| --- | --- |

Equation S7 has 2 main components, on the left side there is the radial integral, henceforth radial matrix element, that can be forthwith calculated with the above shown results, and on the right side, the angular integral, henceforth angular matrix element, that contains an internal product between the polarization vector, ε^(λ)^, and the direction vector, $\hat{r}$. Considering the spherical nature of the problem, this internal product can be expanded to depend on the spherical harmonics.^1^

| $\epsilon\cdot\hat{r}=\sqrt{\frac{4\pi}{3}}\left( \epsilon_{z}Y_{1,0}+\frac{-\epsilon_{x}+i\epsilon_{y}}{\sqrt{2}}Y_{1,1}+\frac{\epsilon_{x}+i\epsilon_{y}}{\sqrt{2}}Y_{1,-1} \right)$ | (S8) |
| --- | --- |

The angular integral is thus divided into 3 different components, according to the incident radiation. One component for the z-polarized radiation, one for the left circularly polarized (LCP) radiation, and one for the right circularly polarized radiation (RCP). Equation S9 shows the resulting expanded angular integral with the 3 components for each polarization.

| $\begin{matrix} \int d\Omega Y_{n_{f}l_{f}}^{\text{*}}(\theta,\phi)\epsilon^{(\lambda)}\cdot\hat{r}Y_{n_{i}l_{i}}(\theta,\phi) & =\epsilon_{z}\sqrt{\frac{4\pi}{3}}\int d\Omega Y_{l_{f},m_{f}}^{\text{*}}Y_{1,0}Y_{l_{i},m_{i}} \\ & +\frac{\epsilon_{x}+i\epsilon_{y}}{\sqrt{2}}\sqrt{\frac{4\pi}{3}}\int d\Omega Y_{l_{f},m_{f}}^{\text{*}}Y_{1,-1}Y_{l_{i},m_{i}} \\ & +\frac{-\epsilon_{x}+i\epsilon_{y}}{\sqrt{2}}\sqrt{\frac{4\pi}{3}}\int d\Omega Y_{l_{f},m_{f}}^{\text{*}}Y_{1,1}Y_{l_{i},m_{i}} \end{matrix}$ | (S9) |
| --- | --- |

As it stands, the above equation coupled with the aforementioned results can be used to calculate the matrix elements. However, the authors preferred to further facilitate the calculation of these elements by applying the following property of the spherical harmonics, as it can also improve the accuracy of the results by avoiding the integration of the spherical harmonics.^3^

| $\left\langle Y_{l_{1}}^{m_{1}} \vert Y_{l_{2}}^{m_{2}} \vert Y_{l_{3}}^{m_{3}} \right\rangle=\sqrt{\frac{\left( 2l_{2}+1 \right)\left( 2l_{3}+1 \right)}{4\pi\left( 2l_{1}+1 \right)}}C\left( l_{2}l_{3}l_{1} \vert000 \right)C\left( l_{2}l_{3}l_{1} \vert m_{2}m_{3}m_{1} \right)$ | (S10) |
| --- | --- |

Where *C(l_2_l_3_l_1_|m_2_m_3_m_1_)* are the Clebsch-Gordon coefficients that are commonly implemented in many programming languages, such as the one used for this work (Python™). As such, the matrix elements can be thus determined and added to Equation 6 of the Main Manuscript to calculate the absorption coefficient for the several transitions.

1. Radial Matrix Elements

In this Section we provide the radial matrix elements, calculated from the radial integral (Equation S7), for the simulated cases provided in the Main Manuscript – 1.6 nm/1.90 eV/0.08 m_e_ (QD radius, potential barrier and effective mass, respectively) in Table S1, 2.5 nm/1.15 eV/0.08 *m_e_* in Table S2 and 2.5 nm/1.90 eV/0.08 *m_e_* in Table S3 - and for the cases provided in Supplementary Material Section S4 – 2.5 nm/1.15 eV/0.08 *m_e_* Table S4 and 3.9 nm/1.15 eV/0.08 m_e_ in Table S5.

Table S1: Table with the radial matrix elements between all possible transitions for the 1.6 nm QD with V_0_ of 1.90 eV and m* of 0.08 m_e_, as calculated from the radial integral in Equation S7.

| **(n, l)** | **0,0** | **0,1** |
| --- | --- | --- |
| **0,0** | – | 0.650 |
| **0,1** | – | – |

Table S2: Table with the radial matrix elements between all possible transitions for the 2.5 nm QD with V_0_ of 1.15 eV and m* of 0.08 m_e_, as calculated from the radial integral in Equation S7.

| **(n, l)** | **0,0** | **0,1** |
| --- | --- | --- |
| **0,0** | – | 1.320 |
| **0,1** | – | – |

Table S3: Table with the radial matrix elements between all possible transitions for the 2.5 nm QD with V_0_ of 1.90 eV and m* of 0.08 m_e_, as calculated from the radial integral in Equation S7.

| **(n, l)** | **0,0** | **0,1** | **0,2** | **1,0** |
| --- | --- | --- | --- | --- |
| **0,0** | – | 1.427 | 1.000 | -0.199 |
| **0,1** | – | – | 1.466 | -0.398 |
| **0,2** | – | – | – | -1.561 |
| **1,0** | – | – | – | – |

Table S4: Table with the radial matrix elements between all the possible transitions for the 2.5 nm QD with V_0_ of 1.15 eV and m* of 0.17 m_e_, as calculated from the radial integral in Equation S7.

| **(n, l)** | **0,0** | **0,1** | **0,2** | **1,0** |
| --- | --- | --- | --- | --- |
| **0,0** | – | 1.445 | 1.165 | -0.052 |
| **0,1** | – | – | 1.555 | -0.592 |
| **0,2** | – | – | – | -1.339 |
| **1,0** | – | – | – | – |

It should be noted that the transitions (*n_i_, l_k_*)→ (*n_i_, l_k_*) are 0 as there cannot be a transition to the same state. Moreover, transitions between two states are independent of the initial state, so that a transition (0, 0)→ (1,0) and (1, 0)→ (0, 0) give the same radial matrix element.

Table S5: Table with the radial matrix elements between all the possible transitions for the 3.9 nm QD with V_0_ of 1.15 eV and m* of 0.17 m_e_, as calculated from the radial integral in equation S7.

| **(n, l)** | **0,0** | **0,1** | **0,2** | **1,0** | **0,3** | **1,1** | **0,4** | **2,1** | **2,0** | **0,5** |
| --- | --- | --- | --- | --- | --- | --- | --- | --- | --- | --- |
| **0,0** | – | 2.251 | 2.130 | -0.599 | 2.124 | -0.045 | 2.135 | 0.246 | 0.000 | 1.869 |
| **0,1** | – | – | 2.552 | -1.205 | 2.357 | -0.322 | 2.428 | 0.053 | -0.105 | 2.532 |
| **0,2** | – | – | – | -1.742 | 2.705 | -0.904 | 2.406 | 0.009 | -0.281 | 2.562 |
| **1,0** | – | – | – | – | -1.774 | 1.854 | -1.611 | 1.069 | -0.066 | -1.948 |
| **0,3** | – | – | – | – | – | -1.519 | 2.771 | -0.631 | 0.285 | 2.324 |
| **1,1** | – | – | – | – | – | – | -1.422 | 1.934 | -1.066 | -1.098 |
| **0,4** | – | – | – | – | – | – | – | -1.392 | 1.053 | 2.767 |
| **2,1** | – | – | – | – | – | – | – | – | -2.213 | -1.086 |
| **2,0** | – | – | – | – | – | – | – | – | – | 0.668 |
| **0,5** | – | – | – | – | – | – | – | – | – | – |

1. Angular Matrix Elements

In this Section we summarize the determined angular matrix elements, as calculated from Equation S7, S9 and S10. Here, we make no distinction regarding the size of the QD as these values depend only on the initial and final (l, m) values. Moreover, as referred in the Main Manuscript, these values were averaged along the allowed *m_i_→m_f_* transitions, since these represent the degeneracy of each level and thus were assumed to have an equal importance in the transition. From Equation S9, it can be seen that the angular matrix elements have 3 components (z, left and right circular polarizations). Table S6 shows the results for the z polarization and Table S7 shows the results for the LCP. The results for the RCP were suppressed as they are equal to those of the left polarization.

Table S6: Table with the angular matrix elements for the z polarization, as determined from Equation S7, S9 and S10.

| **l** | **0** | **1** | **2** | **3** | **4** | **5** |
| --- | --- | --- | --- | --- | --- | --- |
| **0** | 0 | 0.577 | 0 | 0 | 0 | 0 |
| **1** | 0.577 | 0 | 0.470 | 0 | 0 | 0 |
| **2** | 0 | 0.470 | 0 | 0.443 | 0 | 0 |
| **3** | 0 | 0 | 0.443 | 0 | 0.431 | 0 |
| **4** | 0 | 0 | 0 | 0.431 | 0 | 0.424 |
| **5** | 0 | 0 | 0 | 0 | 0.424 | 0 |

Table S7: Table with the angular matrix elements for the left polarization, as determined from Equation S7, S9 and S10.

| **l** | **0** | **1** | **2** | **3** | **4** | **5** |
| --- | --- | --- | --- | --- | --- | --- |
| **0** | 0 | 0.408 | 0 | 0 | 0 | 0 |
| **1** | -0.408 | 0 | 0.315 | 0 | 0 | 0 |
| **2** | 0 | -0.315 | 0 | 0.292 | 0 | 0 |
| **3** | 0 | 0 | -0.292 | 0 | 0.281 | 0 |
| **4** | 0 | 0 | 0 | -0.281 | 0 | 0.275 |
| **5** | 0 | 0 | 0 | 0 | -0.275 | 0 |

1. Absorption for 1.15 eV potential barrier

Here we show the absorption density profiles for QDs with a potential barrier *V_0_=*1.15 eV (e.g. attained with PbS QDs in 1.55 eV bandgap Perovskite host) and a ground-state energy level at -0.90 eV (Figure S1). As such, the QD sizes were determined to be 2.5 and 3.9 nm by a similar procedure to that of Figure 2 of the Main Manuscripts. For these calculations the authors also chose to use a different effective mass (0.17 *m_e_*) since, as referred in the Main Manuscript, there are several references in the literature for PbS’s effective mass with values ranging from 0.08 *m_e_* to 0.17 *m_e_*^4–6^, hence this change provides a broader perspective for the obtained results. The values of the matrix elements necessary for the calculations were provided in the previous sections (S2 and S3).

In general, these results follow the same trend as those of the Main Manuscript, with the notable difference of the higher number of energy levels, especially for the 3.9 nm QD that has 10 energy levels within the potential well. Such a high number of energy levels demonstrates the strong effect of changing the QD radius on the overall QD properties. Nevertheless, the higher number of energy levels ends up also being a massive benefactor on the overall absorption density, as the cumulative absorptions between all levels add up to significantly increase the full absorption magnitude.


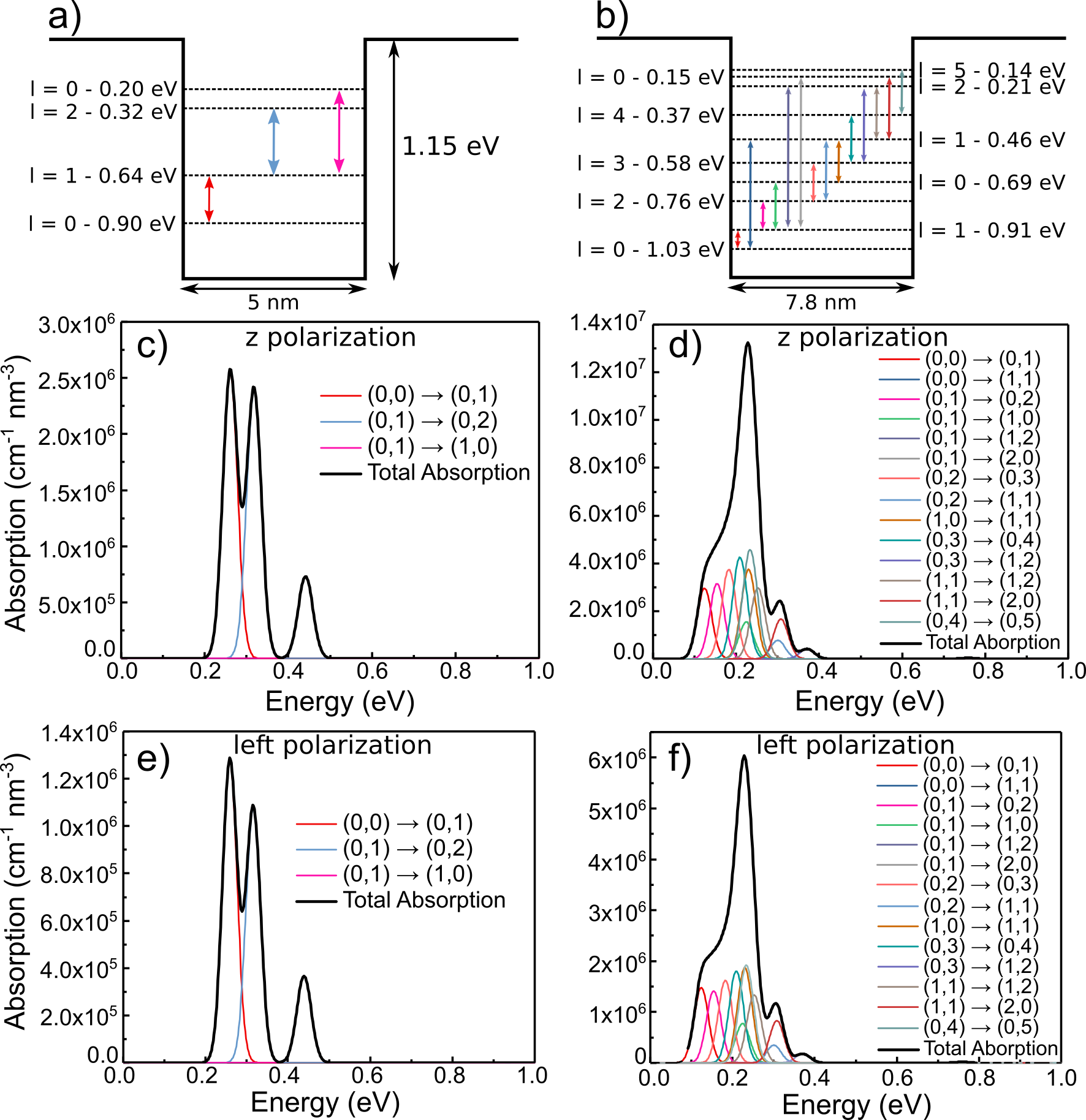


Figure S1: a), b) Energy band diagrams for the 2.5 and 3.9 nm QDs, respectively. The dotted lines in each diagram represent the different energy levels and the colored double-sided arrows the allowed transitions in accordance with the absorption profiles below. Each energy level is also provided together with the specific l value; c), d) absorption density profiles for the z polarization for the 2.5 and 3.9 nm QDs, respectively; e), f) absorption density profiles for the left (or right) polarization for the 2.5 and 3.9 nm QDs, respectively.

References

^1^ S. Gasiorowicz, *Quantum Physics, Third Edition*, 3rd Edition (Wiley, Hoboken, NJ, 2003).

^2^ D.J. Griffiths, *Introduction to Quantum Mechanics*, 2nd Edition (Cambridge University Press, Cambridge, 2016).

^3^ G.B. Arfken, H.J. Weber, and F.E. Harris, *Mathematical Methods for Physicists A Comprehensive Guide.*, 7th ed. (Academic Press, 2012).

^4^ A.K. Walton, T.S. Moss, and B. Ellis, Proc. Phys. Soc. **79**, 1065 (1962).

^5^ J.R. Dixon and H.R. Riedl, Phys. Rev. **140**, A1283 (1965).

^6^ K.K. Nanda, F.E. Kruis, H. Fissan, and S.N. Behera, J. Appl. Phys. **95**, 5035 (2004).
